# Comparison of microglia and infiltrating CD11c^+^ cells as antigen presenting cells for T cell proliferation and cytokine response

**DOI:** 10.1186/1742-2094-11-57

**Published:** 2014-03-25

**Authors:** Agnieszka Wlodarczyk, Morten Løbner, Oriane Cédile, Trevor Owens

**Affiliations:** 1 Department of Neurobiology Research, Institute for Molecular Medicine, University of Southern Denmark, J.B. Winsløwsvej 25, Odense, DK 5000, Denmark

**Keywords:** Antigen presenting cells, Microglia, EAE

## Abstract

**Background:**

Tissue-resident antigen-presenting cells (APC) exert a major influence on the local immune environment. Microglia are resident myeloid cells in the central nervous system (CNS), deriving from early post-embryonic precursors, distinct from adult hematopoietic lineages. Dendritic cells (DC) and macrophages infiltrate the CNS during experimental autoimmune encephalomyelitis (EAE). Microglia are not considered to be as effective APC as DC or macrophages.

**Methods:**

In this work we compared the antigen presenting capacity of CD11c^+^ and CD11c^−^ microglia subsets with infiltrating CD11c^+^ APC, which include DC. The microglial subpopulations (CD11c^−^ CD45^dim^ CD11b^+^ and CD11c^+^ CD45^dim^ CD11b^+^) as well as infiltrating CD11c^+^ CD45^high^ cells were sorted from CNS of C57BL/6 mice with EAE. Sorted cells were characterised by flow cytometry for surface phenotype and by quantitative real-time PCR for cytokine expression. They were co-cultured with primed T cells to measure induction of T cell proliferation and cytokine response.

**Results:**

The number of CD11c^+^ microglia cells increased dramatically in EAE. They expressed equivalent levels of major histocompatibility complex and co-stimulatory ligands CD80 and CD86 as those expressed by CD11c^+^ cells infiltrating from blood. CD11c^+^ microglia differed significantly from blood-derived CD11c^+^ cells in their cytokine profile, expressing no detectable IL-6, IL-12 or IL-23, and low levels of IL-1β. By contrast, CD11c^−^ microglia expressed low but detectable levels of all these cytokines. Transforming growth factor β expression was similar in all three populations. Although CNS-resident and blood-derived CD11c^+^ cells showed equivalent ability to induce proliferation of myelin oligodendrocyte glycoprotein-immunised CD4^+^ T cells, CD11c^+^ microglia induced lower levels of T helper (Th)1 and Th17 cytokines, and did not induce Th2 cytokines.

**Conclusions:**

Our findings show distinct subtypes of APC in the inflamed CNS, with a hierarchy of functional competence for induction of CD4^+^ T cell responses.

## Background

Multiple sclerosis (MS) and its animal model experimental autoimmune encephalomyelitis (EAE) are characterised by infiltration to the central nervous system (CNS) of autoantigen-specific T cells, and recruitment of myeloid cells, including dendritic cells (DC) and macrophages, leading to development of inflammatory lesions, demyelination and axonal damage [1,2]. Recognition of usually myelin epitopes is required for initiation and progression of EAE [1]; however, the mechanisms underlying initiation and control of T cell responses in CNS inflammation are less well understood. Two CD4^+^ T cell subtypes, the IFN-γ secreting T helper (Th)1 and IL-17 secreting Th17 cells, have been shown to play a pathogenic role in EAE [1,3], although neither of the nominal cytokines is absolutely required [4,5].

Activation of CD4^+^ T cells is a multistep process initiated by the appropriate binding of the T-cell receptor to its cognate antigenic peptide presented by major histocompatibility complex (MHC) class II molecules and subsequent stimulation by co-stimulatory molecules such as CD80 and CD86 on the antigen presenting cells (APC). During the activation process, both T cell and APC produce cytokines that shape the immune response. Cytokines that direct Th1 responses include IL-12 and IL-18, while Th17 are directed by transforming growth factor (TGF)-β, IL-1β, IL-6, and IL-23. Regulatory T cells, that exert an anti-inflammatory effect, are directed by TGF-β in the absence of other Th17-inducing signals, or by IL-10 [1].

The APC responsible for T cell re-activation within the CNS remain to be precisely identified. DC, infiltrating macrophages, B cells and CNS-resident microglia can all express MHC class II and co-stimulatory molecules that are required for the initiation and progression of EAE [1]. Of these, DC are the most generally accepted as ‘professional’ APC that can induce an immune response. The fact that DC in the uninflamed CNS are located outside the parenchyma, in perivascular locations [6,7], has contributed to a model whereby T cells receive pro-infiltratory signals through interaction with DC in post-capillary perivascular space [8]. However it remains unclear whether parenchymally located APC, within the CNS, can provide an equivalent signal for infiltrating T cells. Results from a number of studies led to the conclusion that microglia were not as effective APC as DC or macrophages [1,9,10].

We described a subset of CD11c^+^ microglia that were induced in cuprizone-demyelinated or injury-reactive CNS. These cells shared with CD11c^−^ microglia the characteristic of an intermediate level of expression of CD45 that discriminates microglia from blood-infiltrating cells [11]. Phenotypically similar cells were induced by experimental synaptic degeneration in the hippocampus dentate gyrus [12]. Importantly, the cuprizone-induced CD11c^+^ microglia were potent APC for a T cell proliferative response [11].

In this study, we demonstrate that infiltrating CD11c^+^ cells that include DC, and CNS-resident CD11c^+^ microglia sorted from the CNS during EAE and studied directly *ex vivo*, express similar levels of the MHC class I and II molecules, CD80 and CD86. We furthermore show that both populations are equivalently capable of inducing an antigen-specific proliferative response from primed T cells. Whereas infiltrating CD11c^+^ cells expressed all of the Th1- and Th17-inducing cytokines tested, CD11c^+^ microglia only expressed TGF-β and a low level of IL-1β but not IL-6, IL-12p35 or IL-23p19. Interestingly, in contrast to CD11c^+^ microglia, CD11c^−^ microglia did express IL-6, IL-12p35 and IL-23p19. Correspondingly, T cell cytokine responses elicited by these three CNS APC populations differed in magnitude and cytokine profile. CD11c^+^ microglia were weak inducers of Th1 and Th17 cytokines, whereas infiltrating CD11c^+^ cells more strongly induced both Th1 and Th17 cytokines. CNS-resident CD11c^−^ microglia induced very weak proliferative and cytokine responses. Thus, the inflamed CNS contains APC subpopulations with distinct and possibly complementary capability.

## Methods

### Mice

Female C57BL/6j bom (B6) mice aged 6 to 8 weeks were obtained from Taconic Europe A/S, (Lille Skensved, Denmark) and maintained in the Biomedical Laboratory, University of Southern Denmark (Odense). All experiments were approved by the Danish Ethical Animal Care Committee (approval number 2009/561-1724 and 2012-15-2934-00110).

### Active induction of experimental autoimmune encephalomyelitis

Seven- to eight-week-old female mice were immunised by injecting subcutaneously 100 μl of an emulsion containing 100 μg of myelin oligodendrocyte glycoprotein (MOG)_p35–55_ (Department of Biochemistry and Molecular Biology, University of Southern Denmark, Odense) in incomplete freunds adjuvant (DIFCO, Alberstslund, Denmark) supplemented with 400 μg H37Ra *Mycobacterium tuberculosis* (DIFCO). *Bordetella pertussis* toxin (300 ng; Sigma-Aldrich, Brøndby, Denmark) in 200 μl of PBS was injected intraperitoneally at day 0 and day 2. Animals were monitored daily from day 5 and scored on a 6-point scale as follows: 0, no symptoms; 0.5, partial loss of tail tonus; 1, complete loss of tail tonus; 2, difficulty to right, 3, paresis in one or both hind legs; 4, paralysis in one or both hind legs; 5, front limb paresis; 6, moribund. About 75% of the mice showed symptoms of EAE. Severe EAE usually developed 14 to 18 days after immunisation and was defined as a score of 3 to 5.

### Isolation of central nervous system antigen presenting cells, spleen dendritic cells and T cells

To isolate mononuclear cells from the CNS, mice were anaesthetised with 0.2 mg pentobarbital (200 mg/ml; Glostrup Apotek, Glostrup, Denmark) per gram of mouse and intracardially perfused with ice-cold PBS when they showed symptoms of severe EAE. CNS tissue was collected and a single cell suspension was generated by forcing through a 70 μm cell strainer (BD Biosciences, Albertslund, Denmark). Mononuclear cells were collected after centrifugation on 37% Percoll (GE Healthcare Bio-sciences AB, Brøndby, Denmark). They were then first incubated with anti-Fc receptor (Clone 2.4G2; 1 μg/ml; BD Pharmingen,Albertslund, Denmark) and Syrian hamster IgG (50 μg/ml; Jackson Immuno Research Laboratories Inc., Skanderborg, Denmark) in PBS 2% fetal bovine serum (FBS), then with anti-CD45, anti-CD11b and anti-CD11c antibodies (Table 1) in PBS 2% FBS. Cell populations were gated based on isotype control antibodies as CD45^dim^ CD11b^+^ CD11c^−^ (CD11c^−^ microglia), CD45^dim^ CD11b^+^ CD11c^+^ (CD11c^+^ microglia) and CD45^high^ CD11c^+^ and were sorted on a FACSVantage™ or FACSAria™ III cell sorter (BD Biosciences).

**Table 1:** Antibodies used in this study

**Antibody**	**Clone**	**Provider**
PerCP-Cy5.5 -anti-mouse CD11b	M1/70	Biolegend
Biotinylated- anti-mouse CD11c	HL3	BD Pharmingen
FITC- anti-mouse CD80 (B7.1)	16-10A1	Biolegend
FITC- anti-mouse CD86 (B7.2)	GL1	Biolegend
FITC- anti mouse I-A^b^ (MHC class II)	25-9-17	Biolegend
FITC- anti mouse H2-K^b^ (MHC Class I)	AF6-88.5	Biolegend
PE- anti-mouse CD45	30-F11	Biolegend

MHC, major histocompatibility complex. BD Pharmingen, Albertslund, Denmark; Biolegend, Copenhagen, Denmark.

To isolate spleen DC, naive B6 mice were killed by cervical dislocation. Spleens were removed and dissociated by forcing through a 70 μm cell strainer. Erythrocytes were lysed with 0.83% NH_4_Cl. DC were isolated by magnetic separation using CD11c nanobeads (Stemcell Technologies, Grenoble, France). Final purity was 85 to 90%.

T cells for proliferation assay were isolated from mice with severe EAE. Lymph nodes were dissociated by forcing through a 70 μm cell strainer and CD4 T cells were isolated by magnetic separation using a CD4 negative selection kit (Stemcell Technologies). Final purity was 90 to 95% CD4 cells.

### Flow cytometry

Cells were first incubated with anti-Fc receptor and Syrian hamster IgG in PBS, 2% FBS, 0.1% sodium azide then with cell surface marker specific antibodies in PBS, 2% FBS, 0.1% sodium azide as specified in Table 1. Cells stained with biotinylated antibodies were incubated with streptavidin-fluorochrome conjugates (Biolegend, Copenhagen, Denmark). We used the same gating strategy as for fluorescence-activated cell sorting (FACS) and appropriate isotype control antibodies. Data were collected on a FACSCalibur™ or LSRII™ flow cytometer (BD Biosciences) and analyzed using FACSDiva™ software version 6.1.2 (BD Biosciences).

### Proliferation assay

Primed T cells were stained with 2 μM CFSE (Sigma-Aldrich). T cells (2 × 10^5^) from each stained preparation were cultured with CNS APC (5 × 10^4^) with or without 25 μg/ml MOG_p35–55_ peptide in RPMI Glutamax (Invitrogen, Nærum, Denmark) supplemented with 10% FBS, 50 U/ml penicillin-streptomycin (Invitrogen) and 50 μM β-mercaptoethanol (Invitrogen). Supernatants were collected for cytokine assay at day 1. Three days after initiation of the co-culture, cells were harvested, stained with an anti-CD4 antibody and CFSE dilution was analyzed by flow cytometry.

### Cytokine assay

IL-17A and IFN-γ were measured in the culture supernatants from the APC-T-cell cultures using a BD cytometry bead array (CBA) Th1/Th2/Th17 CBA kit following the manufacturer’s instructions (BD Biosciences).

### RNA extraction, quantitative real-time PCR

Sorted CD11c^+^ microglia, CD11c^−^ microglia and CD45^high^ CD11c^+^ cells were placed in RLT buffer (Qiagen, Copenhagen, Denmark) and total RNA was extracted using RNeasy columns as per manufacturer’s protocol (Qiagen). Reverse transcription was performed with M-MLV reverse transcriptase (Invitrogen) according to the manufacturer’s protocol.

Quantitative real-time PCR (qRT-PCR) was performed with 1 μl cDNA in a 25 μl reaction volume containing Maxima® Probe/ROX qPCR Master mix (Fermentas, St Leon-rot, Germany), forward and reverse primers (800 nM; from TAG Copenhagen A/S, Frederiksberg, Denmark) and probe (200 nM; Applied Biosystems, Nærum, Denmark, and TAG Copenhagen A/S). Primer and probe sequences were as follows: CCR2 - Forward: GAAGTATCCAAGAGCTTGATGAAGG; Reverse: CAAGCTCCAATTTGCTTCACAC; Probe: CCACCACACCGTATGACT. TGFβ - Forward: TGACGTCACTGGAGTTGTACGG; Reverse: GGTTCATGTCATGGATGGTGC; Probe: TTCAGCGCTCACTGCTCTTGTGACAG. IL-1β - Forward: CTTGGGCCTCAAAGGAAAGAA; Reverse: AAGACAAACCGTTTTTCCATCTTC; Probe: AGCTGGAGAGTGTGGAT. IL-6 - Forward: TATGAAGTTCCTCTCTGCAAGAGA; Reverse: TAGGGAAGGCCGTGGTT; Probe: CCAGCATCAGTCCCAAGAAGGCAACT. IL-23p19 - Forward: TCTCTGCATGCTAGCCTGGAA; Reverse: ACAACCATCTTCACACTGGATACG; Probe: CGGGACATATGAATCTA. IL-12p35 - Forward: AAGACATCACACGGGACCAAA; Reverse: CAGGCAACTCTCGTTCTTGTGTA; Probe: CAGCACATTGAAGACCTGTTTACCACTGGA.

PCR reactions were done on an ABI Prism 7300 Sequence Detection System (Applied Biosystems). Results were expressed relative to 18S rRNA (2^ΔCT^ method) as endogenous control (TaqMan® Ribosomal RNA control Reagents kit; Applied Biosystems). cDNA was diluted 1/500 for 18S rRNA analysis.

### Statistical analysis

All experiments were repeated at least three times and data are presented as means ± SEM. Statistical significance was assessed using the two-tailed Mann–Whitney U-test (GraphPad Prism 4). *P* values less than 0.05 were considered significant.

## Results

### Different central nervous system antigen presenting cells populations emerge during experimental autoimmune encephalomyelitis

The presence of potential APC in the CNS of both unchallenged and immunised mice was evaluated by their expression of CD11c. Cells were isolated from perfused CNS from either unchallenged B6 mice or from immunised B6 mice with severe EAE. CNS mononuclear cells were analyzed by flow cytometry for expression of CD45 and CD11c. We used relative CD45 levels to discriminate between blood-derived infiltrated (CD45^high^) and resident microglia (CD45^dim^) as previously described [11,12].

**Figure 1:**
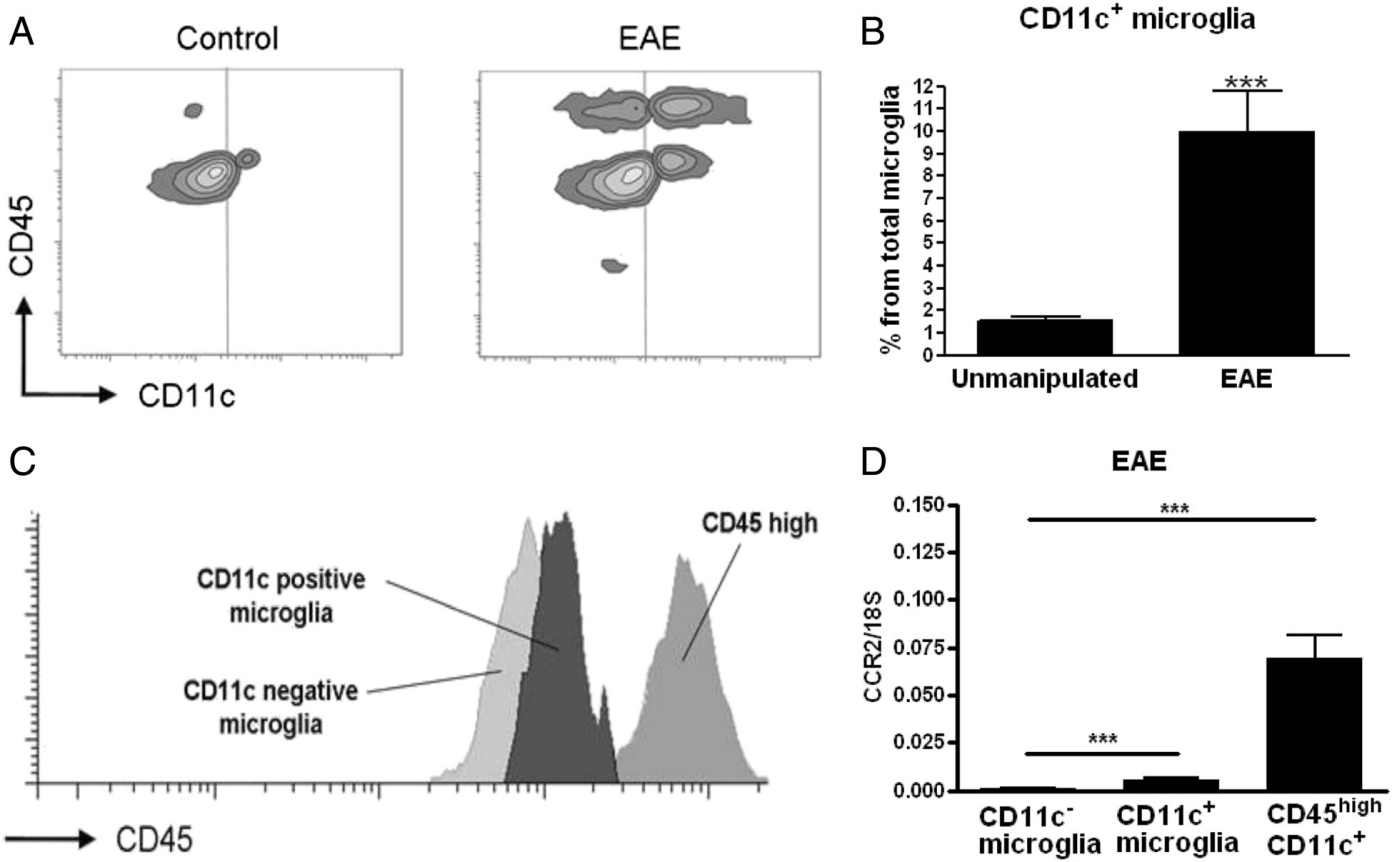
**Comparison of microglia and infiltrating cells in the central nervous system during inflammation.** Representative flow cytometry profiles of individual central nervous system suspensions prepared from control mice or mice with severe experimental autoimmune encephalomyelitis (EAE) showing staining for CD11c and CD45. Vertical line indicates isotype control. Cytometry profile shows increase of both microglia and infiltrating cells expressing CD11c **(A)**. Flow cytometry analysis from six combined experiments shows a significant increase of CD11c^+^ microglia presented as a percentage from total microglia **(B)**. CD11c^+^ microglia showed an increased expression of CD45, but the expression did not reach the level of CD45^high^ expressing cells **(C)**. Quantitative real-time PCR analysis shows significantly lower expression of CCR2 in both population of microglia than in CD45^high^ CD11c^+^ cells **(D)**. Data are presented as a means ± SEM of six individual experiments (n ≥ 13). ***P < 0.001.

We did not detect any CD45^high^ CD11c^+^ cells in CNS of unmanipulated mice. A small population of CD11c^+^ CD45^dim^ microglia was detected (1.7 ± 0.5% of the total microglia population; Figure 1A,B). Immunisation with MOG_p35–55_ resulted in increased proportions of CD11c-expressing cells in the CNS, including an increase in CD11c^+^ microglia (Figure 1A,B) and appearance of blood-derived CD45^high^ CD11c^+^ cells (Figure 1A). Activated microglia have been described to express increased levels of CD45 [13], so it was important to exclude such populations from the cells we analyzed as CD45^high^. We did observe increased CD45 expression on CD11c^+^ microglia compared with microglia that did not express CD11c, but as a population they remained distinguishable from the blood-derived cells that expressed a high level of CD45 (Figure 1C). Recently, resident microglia were described as lacking the chemokine receptor CCR2 and expressing CX3CR1 [14]. We confirmed that CD45^dim^ CD11b^+^ CD11c^+^ cells are CCR2^−^ microglia by qRT-PCR analysis of RNA isolated from FACS-sorted cells. In contrast to CD45^high^ CD11c^+^ cells, neither CD11c^−^ nor CD11c^+^ microglia expressed CCR2 (Figure 1D). Moreover, in CX3CR1 eGFP knockin mice, using a different experimental system, CX3CR1 was expressed by both CD11c^−^ and CD11c^+^ microglia (data not shown).

### CD11c^+^ microglia and CD45^high^ CD11c^+^ cells show similar levels of major histocompatibility complex, CD80 and CD86 expression

**Figure 2:**
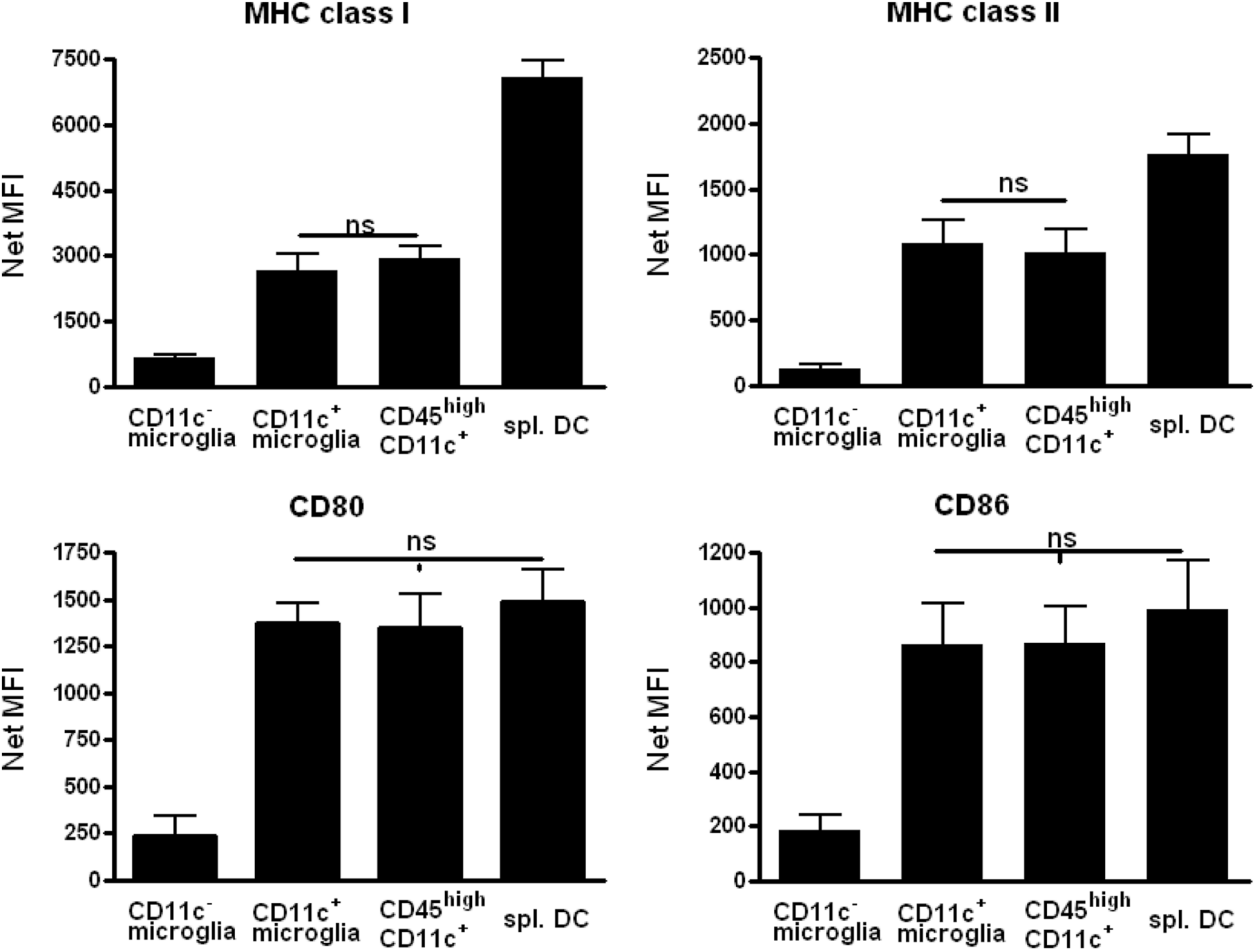
**CD11c**^**+ **^**microglia resemble CD45**^**high **^**CD11c**^**+ **^**cells in expression of molecules needed for antigen presentation and T-cell activation.** Isolated cells from B6 mice with severe experimental autoimmune encephalomyelitis were stained for major histocompatibility complex (MHC) class I, MHC class II, CD80 and CD86 and analyzed by flow cytometry. For comparison of central nervous system antigen presenting cells, cells were gated as CD45^dim^ CD11b^+^, CD11c^−^, CD45^dim^ CD11b^+^, CD11c^+^ and CD45^high^ CD11c^+^. CD11c^+^ cells from the spleen (spl. DC) were used as positive control. Data are presented as means ± SEM of three individual experiments (n = 10). ns, Non-significant differences (*P* > 0.05). NetMFI, Median Fluorescent Intensity – background.

Expression of MHC class II and co-stimulatory molecules CD80 and CD86 is synonymous with the ability to present antigen, and their expression level can be used to indicate APC maturation state. Freshly isolated CD11c^+^ microglia and CD45^high^ CD11c^+^ cells from mice with severe EAE showed equivalent levels of expression of MHC class I and II molecules (Figure 2), but both were significantly lower than on spleen-derived CD11c^+^ cells (Figure 2). Co-stimulatory molecules CD80 and CD86 were likewise expressed at similar levels on CD11c^+^ microglia and CD45^high^ CD11c^+^ cells but, in contrast to MHC class I and II molecules, they were expressed at similar levels as on spleen-derived CD11c^+^ cells (Figure 2). CD11c^−^ microglia expressed significantly lower levels of all of these molecules. Thus, the inflamed CNS contains three potential APC populations, two of which express CD11c and equivalent levels of co-stimulatory molecules to splenic APC.

### CD45^high^ CD11c^+^ cells but not CD11c^+^ microglia express Th1 and Th17 promoting cytokines during experimental autoimmune encephalomyelitis

**Figure 3:**
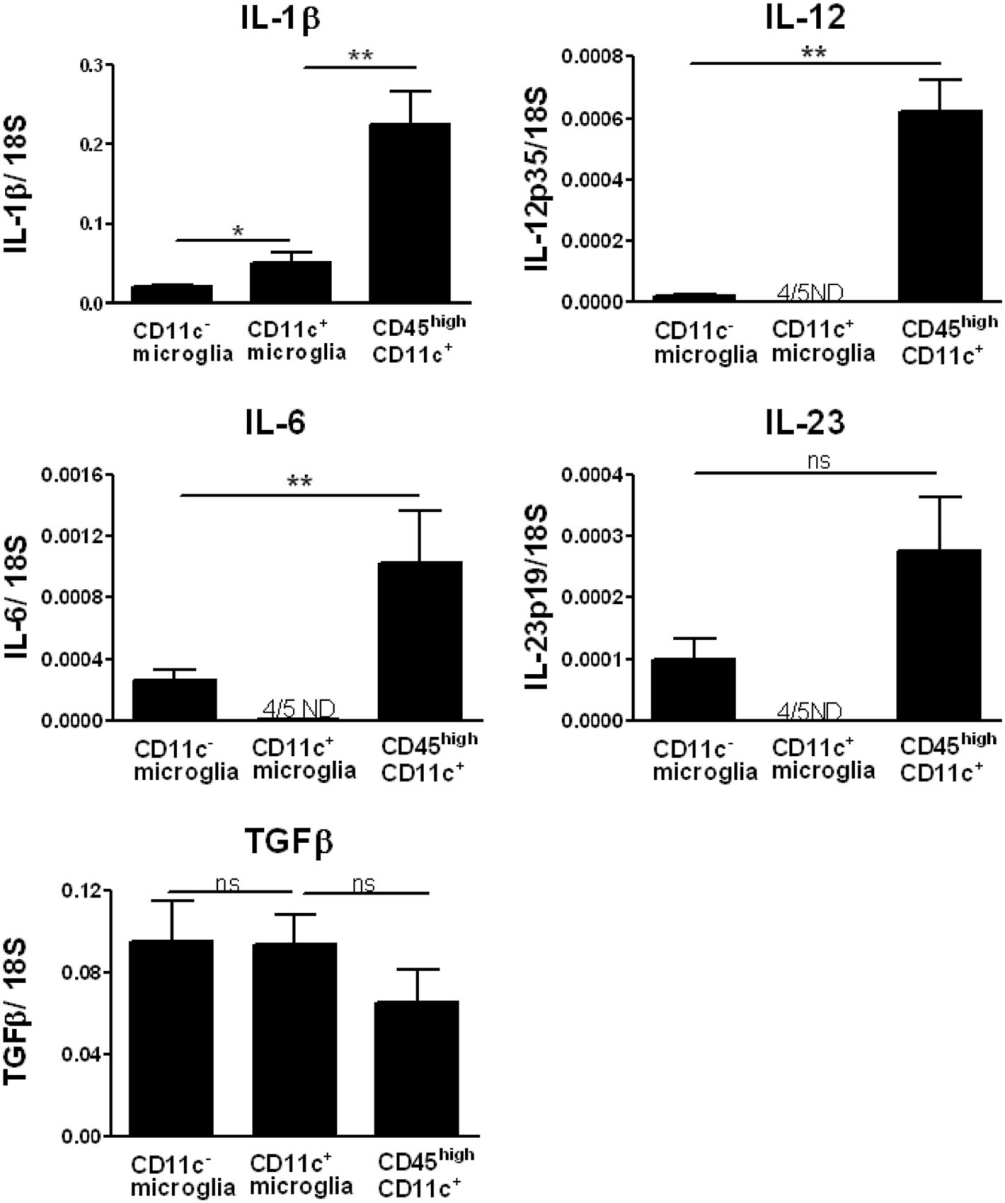
**Cytokine expression profile in antigen presenting cells from the central nervous system of mice with experimental autoimmune encephalomyelitis.** Expression of IL-1β, IL-6, IL-12p35, IL-23p19, and transforming growth factor (TGF)β in fluorescence-activated cell sorted antigen presenting cells (CD11c^+^ microglia, CD11c^−^ microglia and CD45^high^ CD11c^+^) from the central nervous system was analyzed by quantitative real-time PCR. Data are presented as means ± SEM of three individual experiments (n ≥ 5). ns, Not significant; *P < 0.05; **P < 0.01. ND, not detected.

To investigate cytokine expression profile in APC in the CNS of mice with severe EAE we used qRT-PCR to measure transcripts for Th17- and Th1-inducing cytokines in FACS-sorted cells. CD45^high^ CD11c^+^ cells expressed a higher level of the Th17-inducing cytokines IL-1β, IL-6 and IL-23 than microglia; however, all of them expressed similar levels of TGF-β (Figure 3). In contrast to CD11c^−^ microglia, expression of IL-6 and IL-23 in CD11c^+^ microglia was undetectable. CD45^high^ CD11c^+^ cells expressed a higher level of the Th1-promoting cytokine IL-12 than the other cell subsets (Figure 3), and in fact this cytokine was undetectable in CD11c^+^ microglia.

### CD11c^+^ microglia and CD45^high^ CD11c^+^ cells show similar ability to induce proliferation of myelin-specific T cells

**Figure 4:**
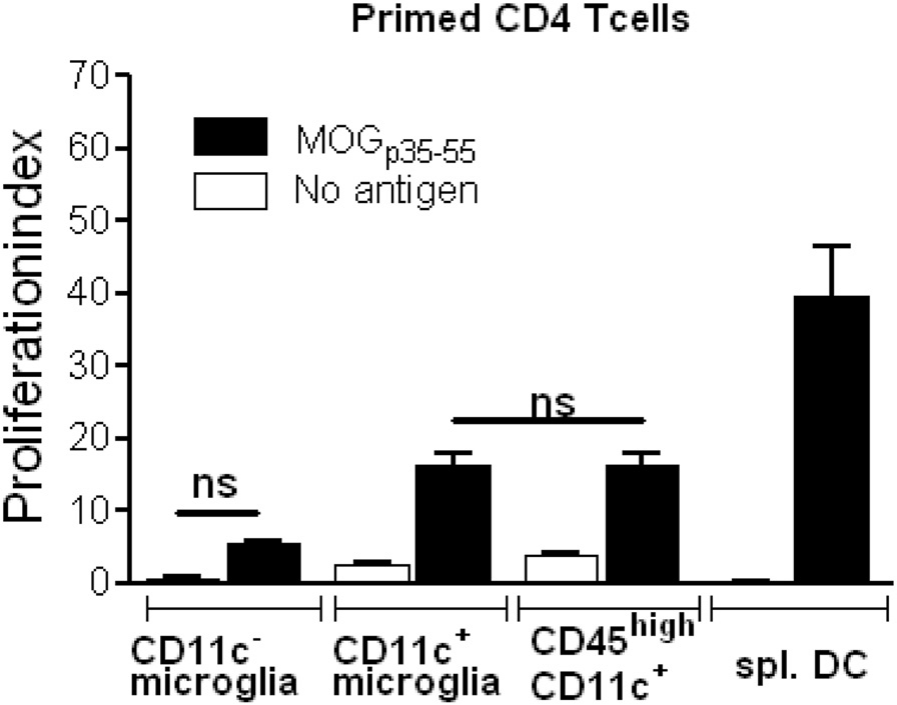
**Relative efficiency of central nervous system and spleen antigen presenting cells in stimulating proliferation of myelin-specific CD4 T cells.** Proliferation of CD4 T cells sorted from B6 mice with severe experimental autoimmune encephalomyelitis (EAE). Splenic CD4^+^ T cells from myelin oligodendrocyte glycoprotein (MOG)_p35–55_ immunised B6 mice with severe EAE were isolated using magnetic beads and cultured with antigen presenting cells (CD11c^−^ microglia, CD11c^+^ microglia, CD45^high^ CD11c^+^ cells and spleen-derived dendritic cells (spl. DC), with (black bars) or without (white bars) MOG_p35–55_. Proliferation was measured by CFSE dilution assay. Data are presented as means ± SEM of three individual experiments (n ≥ 9 ). ns, Non-significant differences (*P* > 0.05).

The ability to reactivate CNS-targeting T cells was evaluated by the induction of proliferative response of MOG-immunised CD4^+^ T cells. CNS APC populations were sorted by flow cytometry. Their ability to induce proliferation in response to MOG_p35–55_ was measured in cultures of CD4^+^ T cells isolated from mice with severe EAE. Both CD11c^+^ microglia and CD45^high^ CD11c^+^ cells induced proliferation of MOG_p35–55_ primed T cells (Figure 4). Proliferative responses induced by CD11c^+^ microglia and CD45^high^ CD11c^+^ cells were similar, though neither of them was comparable to the response to CD11c^+^ cells from spleen (Figure 4). Microglia that did not express CD11c also induced proliferation of primed CD4^+^ T cells, but at a significantly lower level than CD11c^+^ microglia (Figure 4).

### CD11c^+^ microglia induce a distinct cytokine profile in activated T cells

To compare the ability of CD11c^+^ microglia and CD45^high^ CD11c^+^ cells to induce cytokine release when presenting antigen to T cells, we stimulated MOG-immunised CD4^+^ T cells with specific antigen. T cells were cultured with CNS-derived CD11c^+^ microglia, CD11c^−^ microglia, CD45^high^ CD11c^+^ cells or splenic CD11c^+^ cells, in the presence of MOG_p35–55_. After 24 hours, IFN-γ and IL-17A levels in culture supernatants were quantified using CBA.

**Figure 5:**
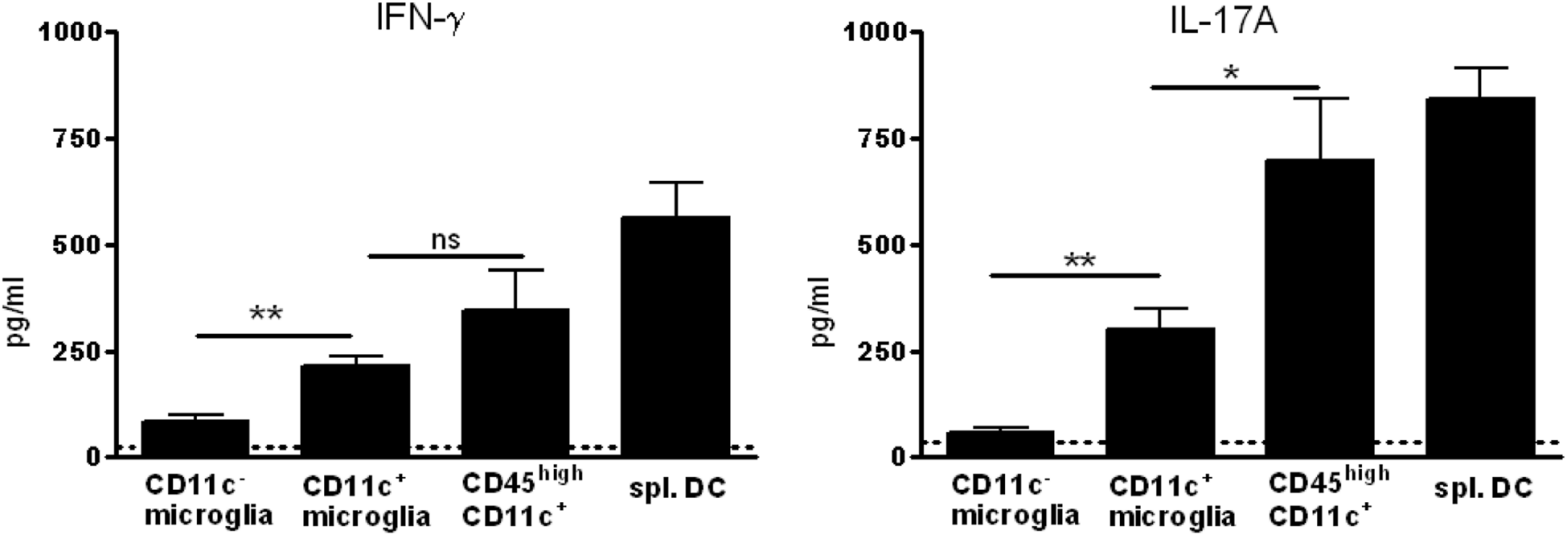
**Cytokine induction by central nervous system antigen presenting cells in primed CD4 T cells.** Fluorescence-activated cell sorted CD4^+^ T cells from myelin oligodendrocyte glycoprotein (MOG)_p35–55_ immunised B6 mice with severe experimental autoimmune encephalomyelitis were co-cultured with antigen presenting cells (CD11c^−^ microglia, CD11c^+^ microglia, CD45^high^ CD11c^+^ cells and spleen-derived dendritic cells (spl. DC) in the presence of MOG_p35–55_ peptide. The cytokines IFN-γ and IL-17A from supernatants were quantified by cytokine bead array. Data are presented as means ± SEM of three individual experiments (n ≥ 9). ns, Not significant; *P < 0.05; **P < 0.01.

CD11c^−^ microglia induced the weakest IFN-γ (Figure 5A) and IL-17A (Figure 5B) response of all the sorted APC populations. CD11c^+^ microglia were much more efficient than CD11c^−^ microglia at inducing release of both cytokines by T cells. Nevertheless they were markedly less effective than CD45^high^ CD11c^+^ cells in this regard (Figure 5). None of the CNS-derived APC were comparable to CD11c^+^ cells derived from spleen in induction of Th1 or Th17 cytokines (Figure 5). As verification that IL17A in the cultures derived from CD4^+^ T cells and not from APC, RT-PCR showed no (microglia) or almost undetectable (CD45^high^ cells) IL17A message in cell-sorted populations from CNS of mice with EAE (data not shown).We also assayed IL-4 and IL-10 in the cultures. Very low levels of those cytokines were induced by all of the APC (data not shown).

## Discussion

We show that three different populations of potential APC can be isolated from the CNS of mice with EAE. These are individual and distinct in terms of their ability to promote CD4^+^ T cell proliferation as well as to induce pro-inflammatory cytokines. Infiltrating CD11c^+^ cells and an inflammation-associated subset of CNS-resident CD11c^+^ microglia show equivalent and potent ability to induce proliferation of antigen-primed CD4^+^ T cells. However they differ in their expression of Th1- and Th17-inducing cytokines and in their quantitative ability to induce such T cell responses, CD11c^+^ microglia being noticeably less effective. In contrast to these, CNS-resident CD11c^−^ microglia express low levels of MHC II and co-stimulatory molecules and are poor inducers of T cell proliferation. Despite higher expression of Th1- and Th17-inducing cytokines than their CD11c^+^ counterparts, they do not induce meaningful Th1 or Th17 responses. The distinct cytokine-producing and response-inducing capabilities of these APC subpopulations identify the complexity of the inflammatory milieu in the CNS in diseases such as MS.

The need for parenchymal APC is based on the fundamental immunological principle of reactivation for CD4^+^ T cell effector function within the target tissue. A role for DC in directing T cell transit from the perivascular space in postcapillary venules has been proposed [10,15]. The possibility that such T cells would exert adequate effector function to induce pathology immediately after crossing the glia limitans cannot be excluded. However, competent microglia are also required for EAE and microglia have been shown to induce final effector CD4^+^ T cell response [9,16]. Furthermore, reactivation of effector function in T cells that migrate deeper than the juxtavascular zone would either require co-infiltrating or already-resident APC. Such considerations motivated our study. Our findings confirm the importance of co-infiltrating CD11c^+^ APC for T cell response in the CNS, but also identify CNS-resident CD11c^+^ APC that can mediate a qualitatively similar outcome.

Despite lack of expression of most of the cytokines that are conventionally associated with Th1 and Th17 induction, CD11c^+^ microglia could nevertheless induce both IFN-γ and IL-17A *in vitro*, although at low levels. IL-17A induction can be explained by the production of TGF-β and IL-1β by CD11c^+^ microglia. Expression of TGF-β was equivalent to that in infiltrating CD11c^+^ populations and levels of IL-1β were likely sufficient to override the induction of regulatory T cells [17]. The induction of a functional Th17 response by IL-1β + TGF-β producing CD11c^+^ microglia that we observed may then reflect the supplementing contribution of IL-6 or IL-23 produced either by other APC contaminating the T cell population, or by *in vitro* induction in microglial APC. IL-12-independent induction of Th1 responses has been described that depends on Type I IFN and IL-18 production by APC [18]. Microglia are known producers of both these cytokines [19-21], so this is a likely explanation for the Th1 responses we observed *in vitro*. Taken together, the observation is that both infiltrating APC and CNS-resident CD11c^+^ microglia can induce Th1 and Th17 responses, but possibly by different routes. How these different routes influence the outcome of CNS inflammation will require increased knowledge of the effect of these induction pathways on the effector CD4^+^ T cell response.

Thus, CD11c^+^ microglia potently induce T cell proliferation but are weak inducers of Th1 and Th17 differentiation, due to lack of expression of necessary cytokines. At the same time, CD11c^−^ microglia are poor inducers of T cell expansion but do produce Th1- and Th17-inducing cytokines. Since both subsets of microglia co-exist during neuroinflammation it is likely that they synergise to drive T cell proliferation and Th1 and Th17 differentiation, and so together may function as equivalently effective CNS-resident APC to the infiltrating CD11c^+^ cells. Emergence of the CD45^dim^CD11c^+^ subset primarily overrides a deficit in induction of T cell expansion.

The infiltrating CD11c^+^ population contained DC. It is noteworthy that their ability to promote T cell proliferation not only was equivalent to that of CD11c^+^ microglia, but that both were markedly less effective than splenic DC-containing populations. Neither DC population was characterised in any more detail and it is possible that this difference reflects varying proportions of other CD11c^+^ cells such as macrophages or granulocytes which produced a quantitative bias. Alternatively, the CNS is known for its immune quiescence, including downregulation of MHC on infiltrating cells [22], and it is possible that the activity of DC that enter the CNS is modulated by the local microenvironment. The relatively high levels of neuronal-derived TGF-β in the CNS have been shown to be responsible for deviation of T cell responses towards a regulatory outcome [23], as well as on microglia [24], and there may be analogous effects on DC.

DC-like CD11c^+^ microglia with neurogenic potential were induced by glatiramer acetate in a transgenic mouse model for Alzheimer’s disease [25]. Other descriptions of phenotypically DC-like microglia in CNS have not discriminated on the basis of relative CD45 levels or other markers that would differentiate them from actual DC or other CD11c^+^ leukocytes [26]. Microglia derive from mesodermal progenitors in the yolk sac that colonise the CNS early in foetal development [27]. They are ontogenically distinct from other mononuclear phagocytic cells, being colony stimulating factor-1-independent [27] and CCR2^−^ CX3CR1^+^[14]. The origin of the CD11c^+^ subset of microglia is of interest. In this study we verified that the two CD11c^+^ populations that emerge in the inflamed CNS in EAE are ontogenically as well as functionally and phenotypically distinct. Our previous data from study of cuprizone demyelination suggested that CD11c^+^ microglia arise through proliferative expansion of a small pre-existing pool [11]. Many studies show an increase of microglia via proliferation during EAE [28-30]. We consider it unlikely that CD11c^+^ microglia increase at the cost of the entire microglia population, but that they are part of a general microglial expansion, although perhaps more favoured under certain circumstances.

The CNS-resident CD11c^+^ microglial subset has by now been described under demyelinating, degenerative and inflammatory conditions and so must be recognised as an important component of innate CNS response.

## Conclusion

Our findings show that the CNS contains different populations of APC during EAE with a hierarchy of functional competence for induction of CD4^+^ T cell responses. The relative activity of these APC reflects lineage as well as local microenvironment. The complexity of the inflammatory milieu in EAE reinforces the uniqueness of the CNS as a site for induction and target of autoimmune response.

## Abbreviations

APC: antigen presenting cells; CBA: cytokine bead array; CNS: central nervous system; DC: dendritic cells; EAE: experimental autoimmune encephalomyelitis; FACS: fluorescence-activated cell sorting; FBS: fetal bovine serum; IFN: interferon; Ig: immunoglobulin; IL: interleukin; MHC: major histocompatibility complex; MOG: myelin oligodendrocyte glycoprotein; MS: multiple sclerosis; PBS: phosphate-buffered saline; qRT-PCR: quantitative reverse-transcriptase polymerase chain reaction; TGF: transforming growth factor; Th: T helper.

## Competing interests

The authors declare that they have no competing interests.

## Authors’ contributions

AW and ML performed the experiments. AW, ML, OC and TO analyzed the data and wrote the paper. All authors read and approved the final manuscript.

## References

[B1] Goverman J Autoimmune T cell responses in the central nervous system Nat Rev Immunol 2009 9 393 407 10.1038/nri2550 19444307 PMC2813731

[B2] McFarland HF Martin R Multiple sclerosis: a complicated picture of autoimmunity Nat Immunol 2007 8 913 919 10.1038/ni1507 17712344

[B3] Becher B Segal BM T(h)17 cytokines in autoimmune neuro-inflammation Curr Opin Immunol 2011 23 707 712 10.1016/j.coi.2011.08.005 21907555 PMC3535446

[B4] Haak S Croxford AL Kreymborg K Heppner FL Pouly S Becher B Waisman A IL-17A and IL-17F do not contribute vitally to autoimmune neuro-inflammation in mice J Clin Invest 2009 119 61 69 19075395 10.1172/JCI35997 PMC2613466

[B5] Popko B Corbin JG Baerwald KD Dupree J Garcia AM The effects of interferon-gamma on the central nervous system Mol Neurobiol 1997 14 19 35 10.1007/BF02740619 9170099 PMC7091409

[B6] McMenamin PG Distribution and phenotype of dendritic cells and resident tissue macrophages in the dura mater, leptomeninges, and choroid plexus of the rat brain as demonstrated in wholemount preparations J Comp Neurol 1999 405 553 562 10.1002/(SICI)1096-9861(19990322)405:4<553::AID-CNE8>3.0.CO;2-6 10098945

[B7] Prodinger C Bunse J Kruger M Schiefenhovel F Brandt C Laman JD Greter M Immig K Heppner F Becher B Bechmann I CD11c-expressing cells reside in the juxtavascular parenchyma and extend processes into the glia limitans of the mouse nervous system Acta Neuropathol 2011 121 445 458 10.1007/s00401-010-0774-y 21076838

[B8] Becher B Bechmann I Greter M Antigen presentation in autoimmunity and CNS inflammation: how T lymphocytes recognize the brain J Mol Med 2006 84 532 543 10.1007/s00109-006-0065-1 16773356

[B9] Ford AL Foulcher E Lemckert FA Sedgwick JD Microglia induce CD4 T lymphocyte final effector function and death J Exp Med 1996 184 1737 1745 10.1084/jem.184.5.1737 8920862 PMC2192872

[B10] Greter M Heppner FL Lemos MP Odermatt BM Goebels N Laufer T Noelle RJ Becher B Dendritic cells permit immune invasion of the CNS in an animal model of multiple sclerosis Nat Med 2005 11 328 334 10.1038/nm1197 15735653

[B11] Remington LT Babcock AA Zehntner SP Owens T Microglial recruitment, activation, and proliferation in response to primary demyelination Am J Pathol 2007 170 1713 1724 10.2353/ajpath.2007.060783 17456776 PMC1854965

[B12] Babcock AA Wirenfeldt M Holm T Nielsen HH Dissing-Olesen L Toft-Hansen H Millward JM Landmann R Rivest S Finsen B Owens T Toll-like receptor 2 signaling in response to brain injury: an innate bridge to neuroinflammation J Neurosci 2006 26 12826 12837 10.1523/JNEUROSCI.4937-05.2006 17151286 PMC6674840

[B13] Carson MJ Reilly CR Sutcliffe JG Lo D Mature microglia resemble immature antigen-presenting cells Glia 1998 22 72 85 10.1002/(SICI)1098-1136(199801)22:1<72::AID-GLIA7>3.0.CO;2-A 9436789

[B14] Mizutani M Pino PA Saederup N Charo IF Ransohoff RM Cardona AE The fractalkine receptor but not CCR2 is present on microglia from embryonic development throughout adulthood J Immunol 2012 188 29 36 10.4049/jimmunol.1100421 22079990 PMC3244524

[B15] Owens T Bechmann I Engelhardt B Perivascular spaces and the two steps to neuroinflammation J Neuropathol Exp Neurol 2008 67 1113 1121 10.1097/NEN.0b013e31818f9ca8 19018243

[B16] Heppner FL Greter M Marino D Falsig J Raivich G Hovelmeyer N Waisman A Rulicke T Prinz M Priller J Becher B Aguzzi A Experimental autoimmune encephalomyelitis repressed by microglial paralysis Nat Med 2005 11 146 152 10.1038/nm1177 15665833

[B17] Jones SA Sutton CE Cua D Mills KH Therapeutic potential of targeting IL-17 Nat Immunol 2012 13 1022 1025 10.1038/ni.2450 23080193

[B18] Freudenberg MA Merlin T Kalis C Chvatchko Y Stubig H Galanos C Cutting edge: a murine, IL-12-independent pathway of IFN-gamma induction by gram-negative bacteria based on STAT4 activation by Type I IFN and IL-18 signaling J Immunol 2002 169 1665 1668 12165484 10.4049/jimmunol.169.4.1665

[B19] Wheeler RD Brough D Le Feuvre RA Takeda K Iwakura Y Luheshi GN Rothwell NJ Interleukin-18 induces expression and release of cytokines from murine glial cells: interactions with interleukin-1 beta J Neurochem 2003 85 1412 1420 10.1046/j.1471-4159.2003.01787.x 12787061

[B20] Khorooshi R Owens T Injury-induced type I IFN signaling regulates inflammatory responses in the central nervous system J Immunol 2010 185 1258 1264 10.4049/jimmunol.0901753 20562259

[B21] Millward JM Lobner M Wheeler RD Owens T Inflammation in the central nervous system and Th17 responses are inhibited by IFN-{gamma}-induced IL-18 binding protein J Immunol 2010 185 2458 2466 10.4049/jimmunol.0902153 20644165

[B22] Hailer NP Glomsda B Blaheta RA Astrocytic factors down-regulate the expression of major histocompatibility complex-class-II and intercellular adhesion molecule-1 on human monocytes Neurosci Lett 2001 298 33 36 10.1016/S0304-3940(00)01711-0 11154829

[B23] Liu Y Teige I Birnir B Issazadeh-Navikas S Neuron-mediated generation of regulatory T cells from encephalitogenic T cells suppresses EAE Nat Med 2006 12 518 525 10.1038/nm1402 16633347

[B24] Abutbul S Shapiro J Szaingurten-Solodkin I Levy N Carmy Y Baron R Jung S Monsonego A TGF-beta signaling through SMAD2/3 induces the quiescent microglial phenotype within the CNS environment Glia 2012 60 1160 1171 10.1002/glia.22343 22511296

[B25] Butovsky O Koronyo-Hamaoui M Kunis G Ophir E Landa G Cohen H Schwartz M Glatiramer acetate fights against Alzheimer’s disease by inducing dendritic-like microglia expressing insulin-like growth factor 1 Proc Natl Acad Sci USA 2006 103 11784 11789 10.1073/pnas.0604681103 16864778 PMC1544247

[B26] Fischer HG Reichmann G Brain dendritic cells and macrophages/microglia in central nervous system inflammation J Immunol 2001 166 2717 2726 11160337 10.4049/jimmunol.166.4.2717

[B27] Ginhoux F Greter M Leboeuf M Nandi S See P Gokhan S Mehler MF Conway SJ Ng LG Stanley ER Samokhvalov IM Merad M Fate mapping analysis reveals that adult microglia derive from primitive macrophages Science 2010 330 841 845 10.1126/science.1194637 20966214 PMC3719181

[B28] McCombe PA de Jersey J Pender MP Inflammatory cells, microglia and MHC class II antigen-positive cells in the spinal cord of Lewis rats with acute and chronic relapsing experimental autoimmune encephalomyelitis J Neuroimmunol 1994 51 153 167 10.1016/0165-5728(94)90077-9 7910169

[B29] Ajami B Bennett JL Krieger C McNagny KM Rossi FM Infiltrating monocytes trigger EAE progression, but do not contribute to the resident microglia pool Nat Neurosci 2011 14 1142 1149 10.1038/nn.2887 21804537

[B30] Ding Z Mathur V Ho PP James ML Lucin KM Hoehne A Alabsi H Gambhir SS Steinman L Luo J Wyss-Coray T Antiviral drug ganciclovir is a potent inhibitor of microglial proliferation and neuroinflammation J Exp Med 2014 211 189 198 10.1084/jem.20120696 24493798 PMC3920559

